# Fabrication of a Ratiometric Fluorescence Sensor Based on Carbon Dots as Both Luminophores and Nanozymes for the Sensitive Detection of Hydrogen Peroxide

**DOI:** 10.3390/molecules27217379

**Published:** 2022-10-30

**Authors:** Yutong Li, Xinhui Gu, Jiayin Zhao, Fengna Xi

**Affiliations:** 1 Department of Chemistry, Key Laboratory of Surface & Interface Science of Polymer Materials of Zhejiang Province, Zhejiang Sci-Tech University, Hangzhou 310018, China; 2 Textile Industrial Products Testing Center of Nanjing Customs District, Wuxi 210001, China

**Keywords:** fluorescent nanozymes, carbon dots, ratiometric fluorescence, fluorescence resonance energy transfer, hydrogen peroxide

## Abstract

The construction of novel fluorescent nanozymes is highly desirable for providing new strategies for nanozyme-based sensing systems. Herein, a novel ratiometric fluorescence sensing platform was constructed based on carbon dots (CDs) as both luminophores and nanozymes, which could realize the sensitive detection of hydrogen peroxide (H_2_O_2_). CDs with peroxidase-mimicking activity were prepared with a one-step hydrothermal method using *L*-histidine as an inexpensive precursor. CDs had bright blue fluorescence. Due to the pseudo-peroxidase activity, CDs catalyzed the oxidation of o-phenylenediamine (OPD) with H_2_O_2_ to generate 2,3-diaminophenolazine (DAP). The fluorescence resonance energy transfer (FRET) between CDs and DAP resulted in a decrease in the fluorescence of CDs and an increase in the fluorescence of DAP, leading to a ratiometric fluorescence system. The free radical trapping experiment was used to investigate the reactive oxygen radicals (ROS) in the catalytic process of CD nanozymes. The enzymatic parameters of CD nanozymes, including the Michaelis constant (*K*_m_) and the maximum initial reaction velocities (*V*_max_), were investigated. A good affinity for both OPD and H_2_O_2_ substrates was proven. Based on the FRET between CDs and OPD, a ratiometric fluorescence analysis of H_2_O_2_ was achieved and results ranged from 1 to 20 μM and 20 to 200 μM with a low limit of detection (LOD, 0.42 μM). The detection of H_2_O_2_ in milk was also achieved.

## 1. Introduction

Natural enzymes are efficient biocatalysts and participate in almost all metabolic processes of living organisms. Due to their high catalytic efficiency and substrate selectivity, natural enzymes have broad application prospects in the fields of industrial production, medical research, bioanalysis, etc. [1]. However, natural enzymes have disadvantages such as high preparation and purification costs, easy denaturation under conditions such as high temperature or extreme pH, and degradation by proteases. In recent years, researchers have been working to develop “artificial enzymes” with enzymatic activity as substitutes for natural enzymes. Amongst researches, nanozymes with the catalytic function of natural enzymes have attracted much attention [2,3,4]. Nanozymes are nanomaterials that have the catalytic ability of natural enzymes and follow the enzymatic characteristics (e.g., catalytic kinetics) of natural enzymes. Due to their excellent properties such as good stability, high catalytic activity, highly tunable structure, and unique properties of nanomaterials, nanozymes are expected to serve as excellent substitutes for natural enzymes in the fields of chemical and biosensing, biomedicine, etc. [5,6,7]. The development of nanozymes with simple preparation methods, low cost, high catalytic performance, and stability is of great significance.

According to their catalytic functions, nanozymes with peroxidase (POD)-, oxidase-, catalase-, and superoxide dismutase (SOD)-mimicking activity have been reported. Among these, POD-mimicking is an important field in nanozyme research. Up to now, researchers have developed POD-mimicking with different structures, including vanadium-based, cobalt-based, or other metal-based nanozymes [8,9,10,11]. However, these materials often suffer from poor biocompatibility, high preparation costs, and complicated preparation processes. Therefore, peroxidase-mimicking nanozymes with simple synthesis, low cost, and good biocompatibility are highly desirable.

Carbon nanomaterials are attractive due to the merits of advantages including their unique structure, multidimensional scale (e.g., zero-dimensional (0D) quantum dots [12,13], 1D nanofibers [14], nanotubes [15,16], nanoribbons [17], 2D nanosheets [18,19,20], and 3D foams [21,22,23,24]), and high biocompatibility. Until now, carbonaceous nanomaterials have been widely used in fields such as energy, catalytic processes, sensing, etc. [25,26,27,28,29]. As a new member of the high value-added nanocarbon family, carbon dots (CDs), also known as carbon quantum dots (CQDs), are a class of 0D carbon nanomaterials with remarkable fluorescence properties [30,31]. Usually, CDs are composed of ultrasmall, dispersed, and quasi-spherical carbon nanoparticles with a size below 10 nm. Due to the quantum confinement effect, CDs have excellent fluorescence properties. In addition, the fluorescence properties of CDs can be flexibly regulated by changing the structure and size of carbon dots [32]. Owing to the merits of good water solubility, environmental friendliness, wide source of synthetic raw materials, and good biocompatibility, CDs have exhibited great potential in medical imaging, environmental monitoring, chemical analysis, energy conversion, and other fields. In recent years, it has also been proven that some CDs have nanozyme activity, which further expands their application scope. Up to now, many synthetic methods have been developed for synthesizing CDs, including the arc discharge method, laser ablation, electrochemical synthesis, hydrothermal synthesis, microwave synthesis, etc. [2,3]. Amongst these, hydrothermal synthesis has the characteristics of simple operation, mild conditions, and high yield. Using inexpensive precursors to synthesize CDs with high catalytic activity through hydrothermal synthesis can greatly expand the application of CD nanozymes.

Until now, most of the existing nanozyme-based sensing systems are based on colorimetric detection. For example, pseudo-peroxidase can catalyze the oxidation of its substrate 3,3′,5,5′-tetramethylbenzidine (TMB) with hydrogen peroxide (H_2_O_2_) [3]. As a result, colorless TMB is oxidized to the oxidation product, oxidized TMB (oxTMB), with a blue color. Thus, the colorimetric detection of oxidizing or reducing substances can be respectively achieved based on the promotion or inhibition of the production of oxTMB. Although colorimetric detection is simple, detection sensitivity still needs to be further improved. Compared with colorimetric detection, fluorescence detection has the significant advantage of high detection sensitivity. Amongst this, ratiometric fluorescence sensing systems perform fluorescence analyses by measuring the intensity of emission spectra at two or more wavelengths. Due to the multiwavelength fluorescence signals, ratiometric fluorescence sensors have the advantages of strong anti-interference ability, high sensitivity, and good reliability, showing great applications in the fields of food safety, environmental pollution analysis, bioanalysis, etc. Fluorescent CDs with nanozyme activity are expected to construct ratiometric fluorescence sensors for reliable and highly sensitive detections.

In this work, carbon dots (CDs) with peroxidase-mimicking activity and blue fluorescence were synthesized through a one-step hydrothermal method using an inexpensive amino acid as a raw material. Owing to the nanozyme activity of CDs, 2,3-diaminophenolazine (DAP) with yellow fluorescence was produced by the catalytic oxidation of o-phenylenediamine (OPD) with hydrogen peroxide (H_2_O_2_). Since the emission spectrum of CDs overlapped with the absorption spectrum of DAP, a fluorescence resonance energy transfer (FRET) occurred with the quenching of the CDs’ fluorescence and the enhancement of the DAP’s fluorescence, leading to a ratiometric fluorescence analysis system. The fabricated ratiometric fluorescence sensor could achieve the sensitive detection of H_2_O_2_ in real food samples.

## 2. Results and Discussion

### 2.1. Synthesis and Characterization of CDs

**Figure 1:**
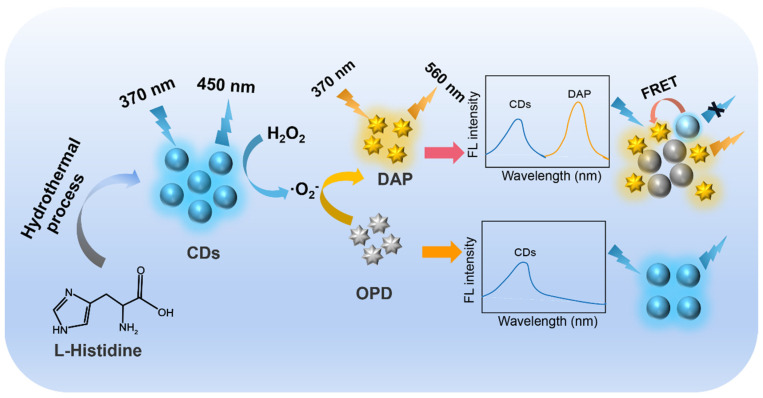
Schematic illustration for the synthesis of CDs and ratiometric fluorescence detection of H_2_O_2_.

Nanomaterials usually exhibit novel optical/electrical/magnetic properties and have a high specific surface area due to their unique microstructure and nanoscale size [33,34,35]. In recent years, the use of various functional nanomaterials to construct novel sensing systems has attracted much attention [36,37,38,39]. As 0D fluorescent carbon nanomaterials, carbon dots (CDs) have the advantages of a wide range of synthetic raw materials and good biocompatibility and have been widely applied in the development of advanced fluorescence sensors. Through changing the precursors and the synthesis conditions, the structure, carbonization degree, conjugated structure, lattice, and energy level structure of CDs can be regulated, leading to highly adjustable fluorescence properties. Among the developed methods for the synthesis of CDs, hydrothermal synthesis usually involves the carbonization or molecular fusion of organic small molecules (precursors) in a hydrothermal reactor at high temperature [40,41,42]. This synthetic method has the advantages of low-cost synthesis and environmental friendliness. It has been proven that the pseudo-peroxidase properties of carbon nanomaterials can be generated through nitrogen doping or the introduction of functional groups (e.g., imidazole) [7,43,44,45]. Inspired by these research works, the amino acid histidine, which was inexpensive and contained an imidazole group, was chosen in this work as the precursor for the synthesis of CDs. As illustrated in Figure 1, CD nanozymes were synthesized with a one-step hydrothermal method. The production yield for the one-step synthesis of CDs was 37.2%.

**Figure 2:**
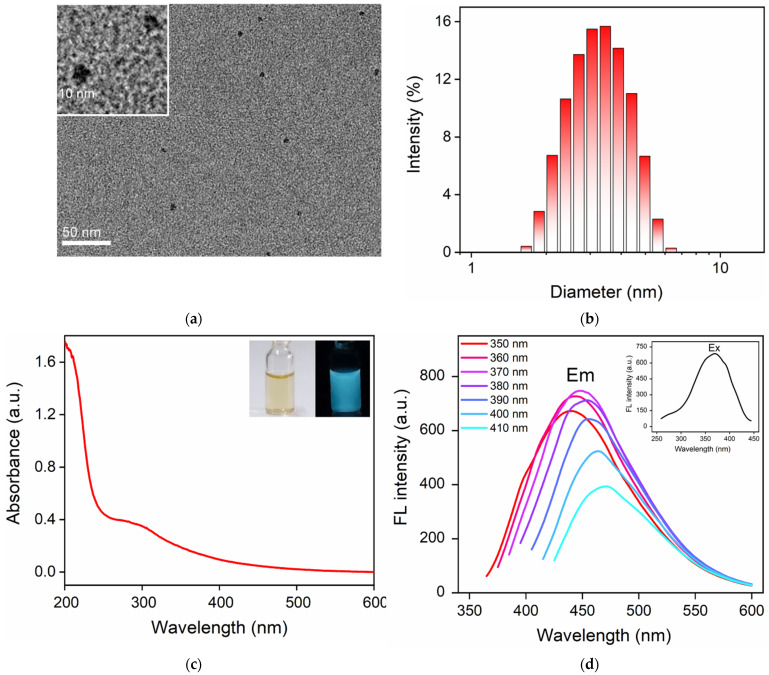
(**a**) TEM image of CDs. The inset is the high-resolution TEM (HRTEM) image. (**b**) The diameter distribution plot of CDs measured with DLS. (**c**) UV absorption spectra of CDs. Insets are optical images of CDs under daylight (**left**) or UV light (**right**, 365 nm). (**d**) The fluorescence (FL) emission spectra (EM) of CDs obtained at different excitation wavelengths ranging from 350 nm to 410 nm (10 nm increments). Inset is the FL excitation spectrum (EX) obtained with an emission wavelength of 450 nm.

The size of CDs was investigated with transmission electron microscopy (TEM). As shown in Figure 2a, CDs had good dispersibility and the particle size varied. The diameter distribution plot of CDs measured with dynamic light scattering (DLS) revealed the average size of 3.2 nm (Figure 2b). Figure 2c shows the UV absorption spectrum of CDs. The absorption peaks at 210 nm and 300 nm corresponded to the π→π * and n→π * transitions. The fluorescence characteristics of CDs were also investigated. The CD solution was light yellow under sunlight, and emitted blue fluorescence under ultraviolet light (365 nm, inset in Figure 2c). As shown in the inset in Figure 2d, the maximum excitation wavelength of CDs was 370 nm. When the excitation wavelength varied from 350 nm to 410 nm, the maximum emission wavelength of CDs slightly changed, indicating an excitation-dependent fluorescence emission. The absolute fluorescence quantum yield was 4.7%.

**Figure 3:**
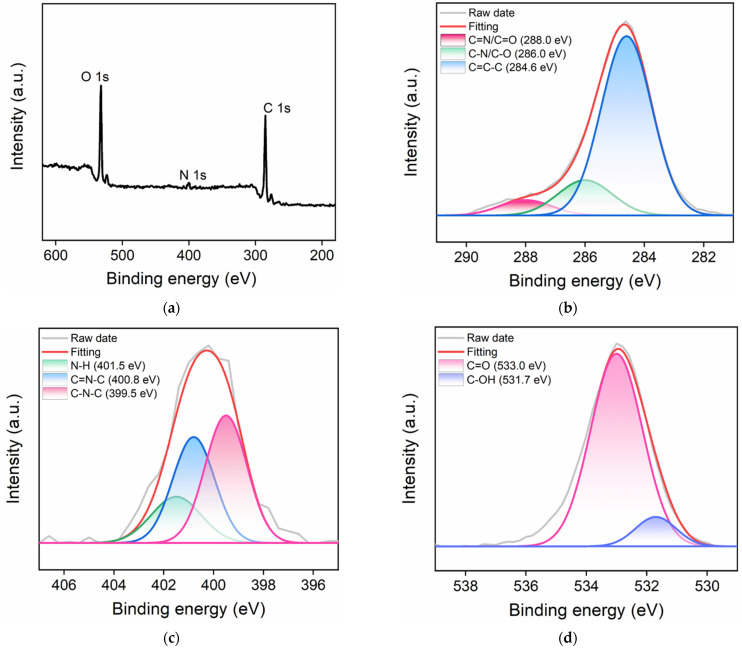
XPS survey spectrum (**a**) and high-resolution C 1s (**b**), N 1s (**c**), and O 1s (**d**) spectra of CDs.

The elemental and chemical compositions of CDs were characterized with X-ray photoelectron spectroscopy (XPS). Figure 3a–d shows the XPS survey spectrum and high-resolution C 1s, N 1s, and O 1s spectra of the prepared CDs. As revealed in the XPS survey spectrum, CDs were composed of three elements including C, N, and O, corresponding to the atomic percentages of 68.5%, 3.3%, and 28.2%, respectively (Figure 3a). This result proved that CDs contained abundant oxygen- and nitrogen-containing groups in addition to the carbon backbone. In the high-resolution C 1s spectrum (Figure 3b), three peaks corresponding to the graphitic C structure (C-C=C, sp^2^ carbon domain), C-N/C-O bond, and C=O/C=N bond were observed. As shown in the high-resolution N 1s spectrum (Figure 3c), the peaks at the binding energies of 399.5 eV, 400.8 eV, and 401.5 eV were related to the C-N-C, C=N-C, and N-H structures, respectively. In addition, the C–OH and C=O structures were proven with the peaks at the binding energies of 531.7 eV and 533.0 eV in the high-resolution O 1s spectrum (Figure 3d). The above results showed that the CDs had imidazole groups and oxygen-containing functional groups such as hydroxyl groups, which were beneficial to the water solubility and nanozyme properties of the CDs.

**Figure 4:**
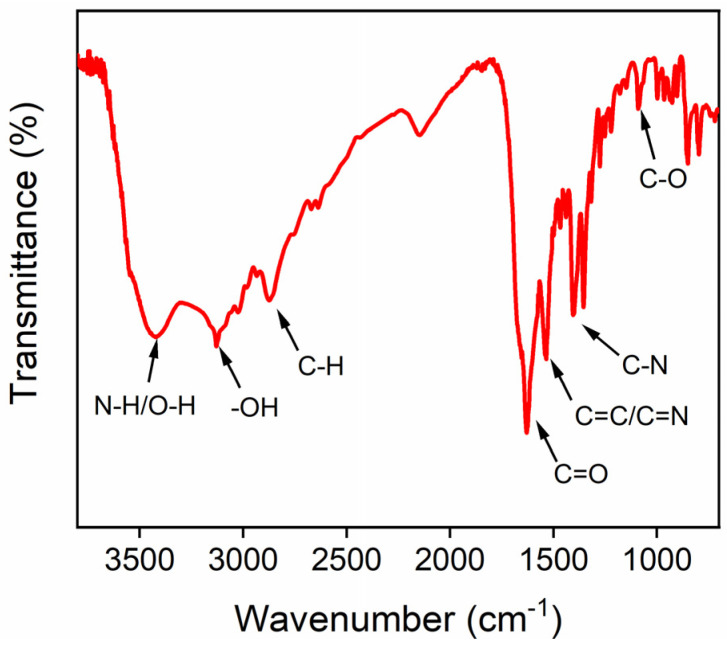
FT-IR spectrum of CDs.

Fourier transform infrared spectroscopy (FT-IR) was used to further characterize the surface functional groups of CDs. As shown in Figure 4, peaks at 3406 cm^−1^, 3128 cm^−1^, and 2860 cm^−1^ were attributed to the stretching vibration of N-H/O-H, -OH, and C-H groups, respectively. In addition, the peaks at 1628 cm^−1^, 1534 cm^−1^, 1357 cm^−1^, and 1093 cm^−1^ were related to the stretching vibrations of C=O, C=C/C=N, C-N, and C-O, respectively. These results proved that there were rich nitrogen and oxygen functional groups on the surface of the CDs.

### 2.2. Peroxidase-Mimicking Activity of CDs

**Figure 5:**
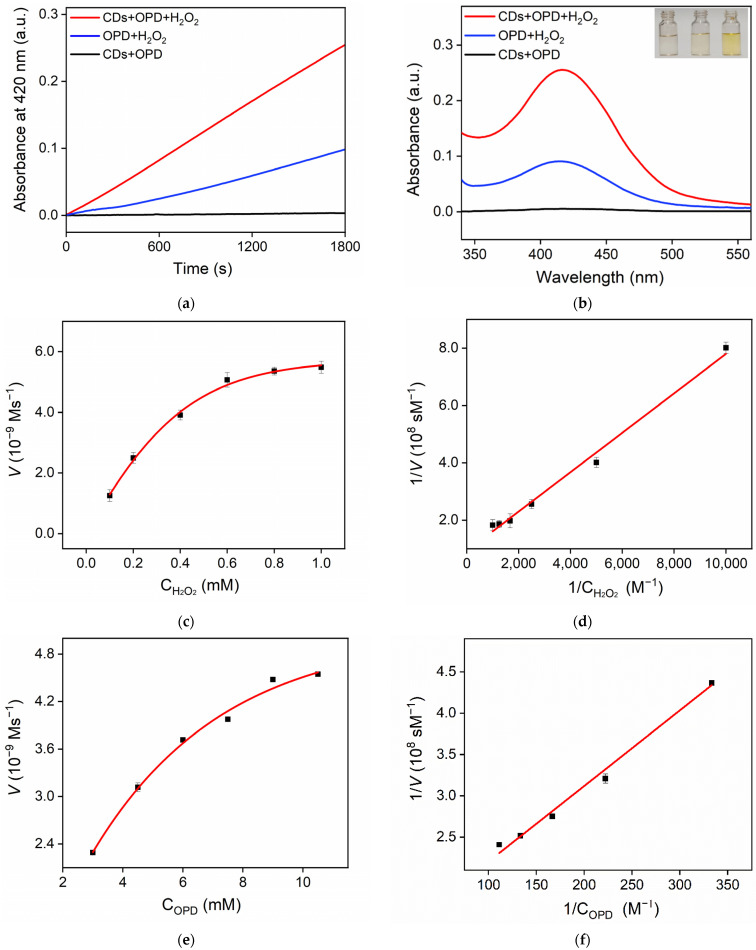
Time-dependent change in A_420_ (**a**) and the corresponding absorbance spectra (**b**) of different mixtures after 35 min reaction. Insets in (**b**) are digital photographs of CDs + OPD (**left**), OPD + H_2_O_2_ (**middle**), and CDs + OPD + H_2_O_2_. (**right**) (**c**,**e**) Steady-state kinetic assay of CDs, in which the reaction velocity was determined through oxidation of OPD based on absorption at 420 nm with varying concentrations of H_2_O_2_ (**c**) or OPD (**e**). (**d**,**f**) Double-reciprocal plots of CD activity obtained using Michaelis–Menten model at different concentrations of H_2_O_2_ (**d**) or OPD (**f**).

Peroxidase is a class of oxidoreductases that use hydrogen peroxide as an electron acceptor to catalyze the oxidation of substrates. Generally, peroxidase catalyzes the oxidation of phenolic, amine, or hydrocarbon compounds with hydrogen peroxide. OPD was chosen as the model substrate to investigate the pseudo-peroxidase activity of the CDs. Generally, CDs catalyze the oxidation of OPD to DAP with H_2_O_2_. DAP is a yellow solution with a characteristic absorption peak at 420 nm. Thus, the enzymatic activity of CDs could be investigated by measuring the characteristic absorption of the reaction system at 420 nm (A_420_). Figure 5a shows the change curve of A_420_ of different mixed solutions with reaction time. As shown, the solution which only contained CDs + OPD basically had no absorption. When OPD was mixed with H_2_O_2_, the absorbance of the solution was lower because H_2_O_2_ could catalyze the oxidation of OPD. However, only a small amount of DAP was produced. When CDs were added to the mixture of OPD and H_2_O_2_, the absorbance value of the CDs + OPD + H_2_O_2_ ternary mixture increased rapidly with the increase in reaction time. Thus, CDs catalyzed the oxidation of OPD with H_2_O_2_, which in turn produced more DAP. Similar results were also observed from the absorbance spectra in Figure 5b. As demonstrated in the digital images (inset of Figure 5b), the OPD + CDs + H_2_O_2_ solution had a deep yellow color, proving that the presence of CDs produced the most DAP. These phenomena demonstrated the peroxidase-mimicking activity of the CDs, which catalyzed the oxidation of OPD with H_2_O_2_.

### 2.3. Steady-State Dynamics of CD Nanozymes

As the peroxidase mimicked, the steady-state kinetic constants of CD nanozymes were investigated to evaluate their enzymatic properties. The double-reciprocal curve of the Michaelis equation (Lineweaver–Burk plots, 1/*V*_0_ = *K*_m_/*V*_max_ *C* + 1/*V*max) was used to analyze the Michaelis constant (*K*_m_) and the maximum reaction rate (*V*_max_). The Michaelis–Menten curve was measured with H_2_O_2_ and OPD as substrates, respectively (Figure 5c,e). According to the Lineweaver–Burk curve, *K*_m_ was calculated to be 0.7468 mM and the *V*_max_ was 1.086 × 10^−8^ m/s with H_2_O_2_ as the substrate (Figure 5d). When OPD was applied as the substrate, the calculated *K*_m_ and *V*_max_ were 6.81 mM and 7.60 × 10^−9^ m/s, respectively (Figure 5f). The small *K*_m_ proved high affinity towards the substrates. The *K*m with OPD as the substrate was lower than that obtained on Fe-doped carbon dots (Fe-CDs) [46] and mesoporous MnCo_2_O_4_ [47]. The *K*_m_ with H_2_O_2_ as the substrate was lower than that obtained on Co- and N co-doped carbon dots (Co, N/CDs) [48], ruthenium ion-complexed carbon nitride (Ru-C_3_N_4_) [49], and horseradish peroxidase (HRP) [50].

### 2.4. Catalytic Mechanism of CD Nanozymes

**Figure 6:**
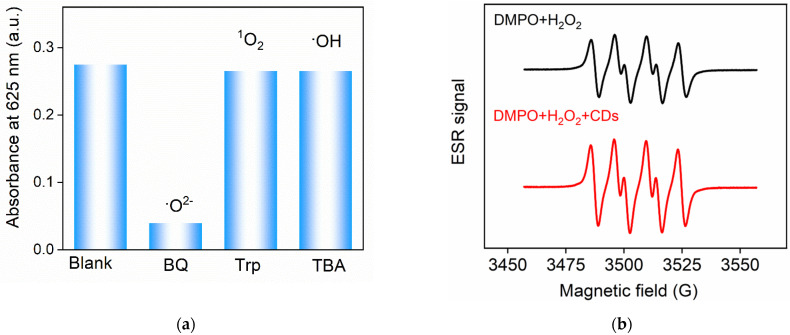

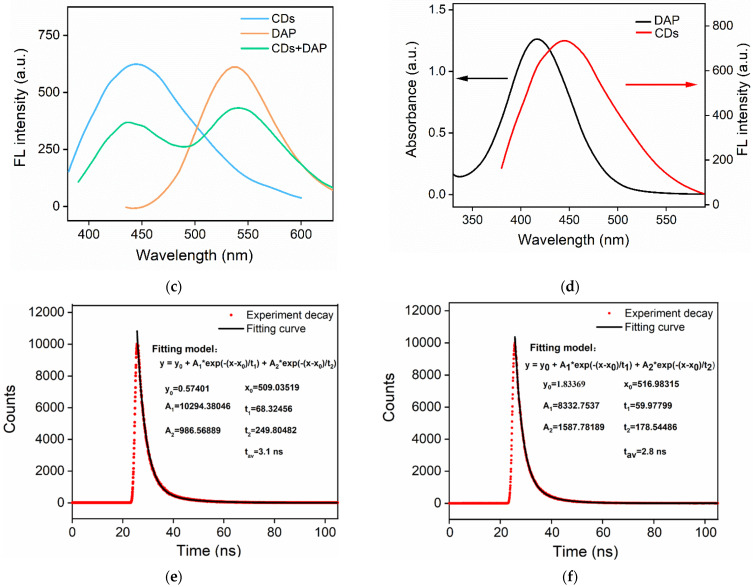
(**a**) A_652_ obtained in the absence (blank) or presence of the capture agent of ROS. (**b**) ESR spectra obtained in the mixture of DMPO and H_2_O_2_ in the absence or presence of CDs. (**c**) Fluorescence emission spectra of CDs (15 μg/mL), DAP (0.5 mM), and CDs (15 μg/mL) + DAP (formed in the presence of 50 μM H_2_O_2_ and 7.5 mM OPD). (**d**) The absorbance spectra of DAP and the fluorescence emission spectrum of CDs. (**e**,**f**) Fluorescence lifetime spectra of CDs in the absence (**e**) or presence of DAP (**f**).

To identify the types of reactive oxygen species (ROS) produced in the CD-catalyzed process, experiments to capture the ROS were carried out [9]. TMB was applied as the substrate and the oxidation of TMB with H_2_O_2_ catalyzed with CDs was studied. 1,4-benzoquinone (BQ) was used as the capture agent of the superoxide anion radical (•O^2−^), tryptophan (Trp) was employed to capture singlet oxygen (^1^O_2_), and tert-butanol (TBA) was used as the capture agent of •OH. When free radicals were captured, the oxidation process of TMB was inhibited, resulting in a lower amount of oxidized TMB and a low characteristic absorption at 652 nm. As shown in Figure 6a, the absorbance of the system with BQ decreased significantly, proving that the ROS produced in the catalytic process of CDs was mainly •O^2−^. These conclusions were further verified with electron spin resonance (ESR) spectra in Figure 6b. An approximately 1:1:1:1 peak appeared in the mixed solution of DMPO and H_2_O_2_, which was the characteristic peak of •O^2−^ resulting from the decomposition of H_2_O_2_. When CDs were added, the signal of •O^2−^ was significantly enhanced, indicating that CDs promoted the H_2_O_2_ decomposition to generate more •O^2−^. Thus, CDs catalyzed the formation of •O^2−^, facilitating the oxidation of the substrate.

### 2.5. FRET Phenomenon between CDs and DAP

A fluorescence resonance energy transfer (FRET) is a special phenomenon that occurs directly between two fluorescent substances. The occurrence of this phenomenon requires two prerequisites. One is that the emission spectrum of one fluorescent material has a certain overlap with the absorption spectrum of another material. Second, the distance between the two fluorophores has to be relatively close. When these two conditions are satisfied at the same time, the phenomenon of fluorescence energy transfer from the donor to the acceptor can be observed. As shown in Figure 6c, CDs had blue fluorescence and DAP emitted yellow fluorescence. In the mixture of CDs and DAP, a decrease in the CDs’ fluorescence was observed, indicating the potential of the FRET phenomenon. As shown in Figure 6d, the absorption spectrum of DAP and the fluorescence emission spectrum of CDs had obvious overlap. The fluorescence lifetime of CDs in the presence or absence of DAP was further investigated. As shown, after the addition of DAP, the fluorescence lifetime of CDs decreased from the original 3.1 ns (Figure 6e) to 2.8 ns (Figure 6f), proving that the presence of DAP led to the dynamic quenching of the CDs’ fluorescence. These results indicated the FRET between the CDs and DAP.

### 2.6. Ratiometric Fluorescence Detection of H_2_O_2_

H_2_O_2_ is an important metabolite in cells and a signaling molecule related to oxidative stress and physiological activities. The accumulation of H_2_O_2_ can damage DNA, protein, and lipid chains in cells and lead to accelerated apoptosis of cells. An abnormal H_2_O_2_ concentration is considered to be a direct trigger of various physiological diseases, including autoimmune diseases, Alzheimer’s disease, and cancer. Therefore, the detection of H_2_O_2_ is of great significance in biomedical research, clinical diagnosis, food safety, etc. In this work, a ratiometric fluorescence assay was constructed for the simple, rapid, and highly sensitive detection of H_2_O_2_. As illustrated in Figure 1, a ratiometric fluorescence system to detect H_2_O_2_ was constructed since CDs and DAP could directly undergo the FRET. On the one hand, CD nanozymes catalyzed the decomposition of H_2_O_2_ to generate •O^2−^, and •O^2−^ oxidized OPD to reduce DAP. On the other hand, the FRET occurred between DAP and CDs, resulting in a decrease in the fluorescence intensity of CDs at 450 nm and an increase in the fluorescence intensity of DAP at 560 nm. Thus, a dual signal change in the fluorescence emission was observed. Based on this ratiometric fluorescence signal, the detection of H_2_O_2_ could be achieved.

**Figure 7:**
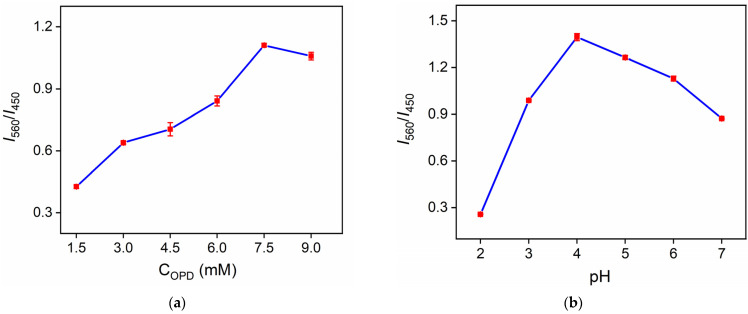

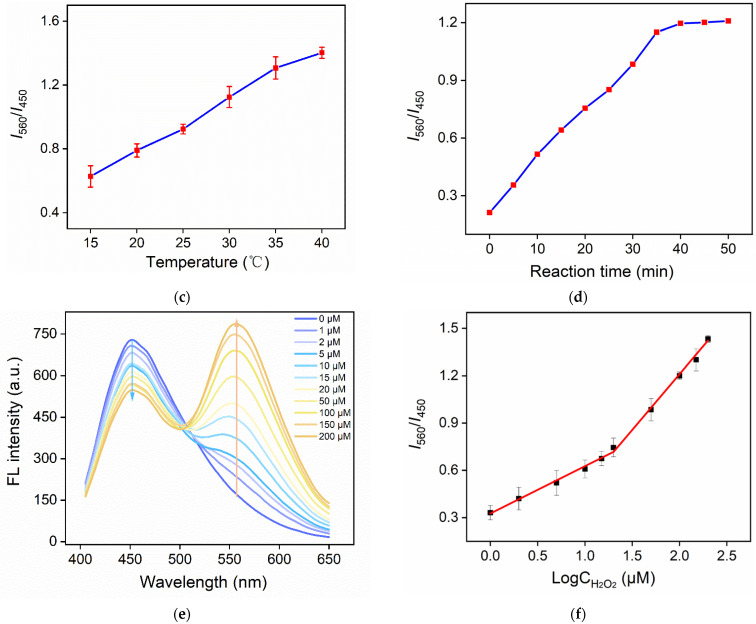
The effect of OPD concentration (**a**), pH (**b**), temperature (**c**), and reaction time (**d**) on *I*_560_/*I*_450_ signals. *I*_560_ and *I*_450_ represent fluorescence intensity at 560 nm or 450 nm, respectively. (**e**) The fluorescence emission spectra of OPD + CDs solution in the presence of different concentrations of H_2_O_2_. The excitation wavelength was 390 nm. (**f**) The regression curve between I_560_/I_450_ and the logarithmic value of hydrogen peroxide concentration.

To obtain the best conditions for the detection of H_2_O_2_, the detection parameters including the concentration of OPD and the reaction conditions (pH, temperature, and reaction time) were optimized (Figure 7). The effects of the above parameters were investigated using the ratio of the fluorescence intensity at 560 nm to the fluorescence intensity at 450 nm (*I*_560_/*I*_450_) as the signal. The increase in the substrate of OPD firstly led to the increase in *I*_560_/*I*_450_ resulting from the formation of DAP (Figure 7a). Then, a reaction plateau was achieved. It was found that *I*_560_/*I*_450_ had a maximum value at pH = 4.0, which proved that the CD nanozymes had the highest reactivity (Figure 7b). This was possibly due to the high reactivity of H_2_O_2_ at this pH. When the reaction temperature was 40 °C, *I*_560_/*I*_450_ had a maximum value, proving that the CD nanozymes had the highest reactivity close to the physiological temperature like natural enzymes (Figure 7c). In addition, the reaction reached equilibrium at 35 min (Figure 7d). These optimized conditions were used for further experiments.

Figure 7e shows the fluorescence spectra after adding different concentrations of H_2_O_2_ to the CDs + OPD system. As can be seen, with the increase in H_2_O_2_ concentration, the fluorescence intensity of CDs at 450 nm decreased, and the fluorescence intensity of DAP at 560 nm increased. A linear relationship was observed between *I*_560_/*I*_450_ and the logarithmic value of the hydrogen peroxide concentration (log*C*_H__2O__2_) at 1–20 μM (*I*_560_/*I*_450_ = 0.326logC_H__2O__2_ + 0.300, *R*^2^ = 0.991) and 20–200 μM (*I*_560_/*I*_450_ = 0.707logC_H__2O__2_–0.204, *R*^2^ = 0.994) (Figure 7f). The detection limit calculated based on the signal-to-noise ratio of three (S/N = 3) was 0.42 μM. The comparison between the determination of H_2_O_2_ using different nanomaterials and methods is demonstrated in Table 1 [47,49,51,52,53,54]. The LOD was lower than that obtained using iron-doped CuSn(OH)_6_ microspheres (Fe/CuSn(OH)_6_) [51], FePt-Au ternary metallic hybrid nanoparticles (Fe Pt/AuHNPs) [52], mesoporous MnCo_2_O_4_ [47], Ru/C_3_N_4_ [49], Fe-doped carbon dots immobilized on cellulose nanofibrils (CNF/FeCDs) [53], and zinc and nitrogen co-doped multifunctional carbon dots (N,Zn/CDs) [54].

### 2.7. The Selectivity of the Detection and Real Sample Analysis

**Figure 8:**
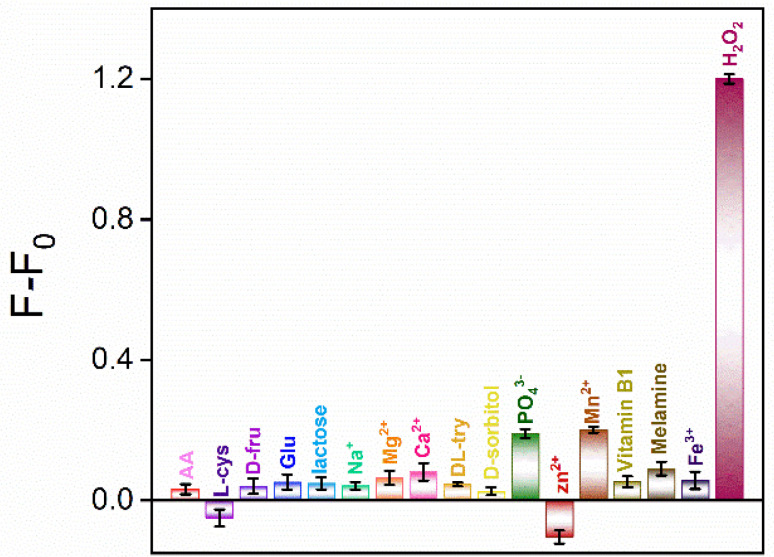
The change in fluorescence intensity ratioof OPD + CDs solution in the absence (F_0_) or presence (F) of different substances. F and F_0_ were calculated using the fluorescence intensity (*I*) at 560 nm and 450 nm (F = *I*_560_/*I*_450_) in each reaction system, respectively. The concentrations of H_2_O_2_, Fe^3+^, and other substances were 0.2 mM, 0.6 μg/mL, and 4 mM, respectively.

Selectivity is important for the application of the sensor. To evaluate the detection selectivity of the constructed ratiometric fluorescence sensor using CDs as luminophores and nanozymes, the effect of ascorbic acid (AA), *L*-cysteine, *D*-fructose (*D*-fru), glucose (Glu), lactose, Na^+^, Mg^2+^, Ca^2+^, *D*,*L*-tryptophan (*D*,*L*-try), D-sorbitol, PO_4_^3−^, Zn^2+^, Mn^2+^, vitamin B1, melamine, and Fe^3+^ was investigated. Figure 8 shows the fluorescence intensity ratioof the OPD + CDs solution in the absence (F_0_) or presence (F) of different substances, where F or F_0_ were calculated using the fluorescence intensity (*I*) at 560 nm and 450 nm (F = *I*_560_/*I*_450_). As shown, in the presence of H_2_O_2_, the signal increased significantly. In contrast, when the above substances were added, there was no obvious change in *I*_560_/*I*_450_, indicating that the ratiometric fluorescence sensor had good selectivity.

The H_2_O_2_ in milk was detected with a standard addition method. As shown in Table 2, a good recovery, ranging from 97.0% to 102%, was obtained when the constructed ratiometric fluorescence sensor was applied to detect H_2_O_2_. The relative standard deviation (RSD) of the determination was less than 2.9%, proving good reliability.

## 3. Materials and Methods

### 3.1. Chemicals and Materials

*L*-histidine was obtained from Shanghai Kangjie Biological Technology Development Co., Ltd. (Shanghai, China). Hydrogen peroxide (H_2_O_2_) and zinc chloride (ZnCl_2_) were provided by Tianjin Yongda Chemical Reagent Co., Ltd. (Tianjin, China). Tryptophan (Trp), 1,4-benzoquinone (BQ), tert-butanol (TBA), o-phenylenediamine (OPD), ascorbic acid (AA), *L*-cysteine, *D*-fructose, glucose, D-sorbitol, melamine, vitamin B1, lactose, trisodium phosphate anhydrous (Na_3_PO_4_), sodium chloride (NaCl), iron chloride (FeCl_3_), magnesium sulfate (MgSO_4_), Manganese acetate tetrahydrate (Mn(CH_3_COO)_2_·4H_2_O), calcium chloride (CaCl_2_), *D*, *L*-tryptophan, and 3,3′,5,5′-tetramethylbenzidine dihydrochloride (TMB) were purchased from Aladdin Reagent Company (Shanghai, China). Milk was purchased from a local supermarket (Hangzhou, China).

### 3.2. Experiments and Instrumentations

The size of the CDs was characterized using a JEM-2100 transmission electron microscopy (TEM) with an ultrathin carbon film as the supporting substrate. The applied voltage was 200 kV. The dynamic light scattering (DLS) was measured using a SZ-100V2 nanoparticle analyzer (HORIBA, Kyoto, Japan) equipped with a 633 nm He–Ne laser. The scattering light at a 90 angle was detected and used to analyze the size and size distribution. The chemical composition of CDs was analyzed with X-ray photoelectron spectroscopy (XPS, PHI5300, PE, Waltham, MA, USA) using Mg Kα radiation (250 W, 14 kV). UV–Vis absorption spectra of the CDs were recorded using a UV-2450 UV–Vis spectrophotometer (Shimadzu, Japan). The fluorescence properties of CDs were investigated using a Fluoromax-4 spectrophotometer (Horiba, Montpellier, France). Electron paramagnetic resonance (EPR) spectra were obtained using an EMX-10/12 spectroscopic instrument (Bruker, Karlsruhe, Germany). Fourier transform infrared spectroscopy (FT-IR) was measured using a Vertex 70 spectrometer (Bruker, Karlsruhe, Germany) at room temperature on power-pressed KBr pellets.

### 3.3. Synthesis of CDs

CDs were synthesized with a one-step hydrothermal method. Specifically, 0.31 g of *L*-histidine was added into 20 mL of deionized water. Then, the mixture was added into a Teflon-lined autoclave and reacted at 180 °C for 10 h. The obtained solution was filtered with a filter film (0.22 μm) to remove large-sized particles, and the filtered solution was dialyzed at room temperature in a dialysis bag with a cut-off molecular weight of 500 Da for 72 h to remove unreacted small molecules or salts generated by the reaction. The dialysate was freeze-dried to obtain CD solids.

### 3.4. Peroxidase-Mimicking Activity of CDs

OPD was selected as the substrate to investigate the peroxidase-mimicking activity of CDs. The absorbance spectrum of the oxidized product, DAP, generated by the CD-catalyzed oxidation of OPD with H_2_O_2_ was determined. Briefly, a Tris-HCl buffer solution (Tris-HCl, 50 mM, pH = 4.0) was used as the medium. Three samples including the control groups (OPD + H_2_O_2_ and OPD + CDs) and the experimental group (OPD + H_2_O_2_ + CDs) were investigated. The absorbance spectra were measured after 35 min of reaction at 40 °C. The used concentrations of OPD, H_2_O_2_, and CDs were 7.5 mM, 0.2 mM, and 15 μg/mL, respectively.

To investigate the catalytic mechanism of CD nanozymes, the trapping of free radicals generated during the catalytic process was investigated. TMB was applied as the substrate of peroxidase-mimicking nanozymes and CDs (15 μg/mL), and in the catalyzed oxidation of TMB, hydrogen peroxide was used as the model. P-benzoquinone, tryptophan, and tert-butanol were chosen as the scavengers for the superoxide anion radical (•O^2−^), singlet oxygen (^1^O_2_), and hydroxyl radical (•OH). Specifically, one of the above three capture agents (100 μM for p-benzoquinone and 100 μg/mL for both tryptophan and tert-butanol) was added to the TMB (0.5 mM) + CDs (15 μg/mL) + H_2_O_2_ (3.3 mM) system and the absorbance value of the solution at 652 nm (A_652_) was measured after 10 min of reaction.

The ESR test was further carried out using 5,5-dimethyl-1-pyrroline-N-oxide (DMPO) as a trapping agent to determine the amount of •O^2−^ generated by the catalytic reaction. Experiments were performed in methanol solution (pH = 4.0). DMPO + H_2_O_2_ and DMPO + H_2_O_2_ + CDs solutions were respectively prepared and then exposed to UV light for 2 min. The concentrations of DMPO, H_2_O_2_, and CDs were 20 mM, 200 mM, and 30 μg/mL, respectively.

### 3.5. Ratiometric Fluorescence Detection of H_2_O_2_ and Real Sample Analysis

For the detection of H_2_O_2_, OPD (7.5 mM), and CDs (15 μg/mL), a Tris-HCL (50 mM, pH = 4.0) was used as the detection medium. When different concentrations of H_2_O_2_ were added, the mixture was incubated in the dark at 40 °C for 35 min. Then, the emission spectrum of the solution was recorded with an excitation wavelength of 390 nm. To investigate the selectivity of the constructed ratiometric fluorescence sensor for detecting H_2_O_2_, ascorbic acid (AA), D-sorbitol, melamine, vitamin B1, *L*-cysteine, *D*-fructose, glucose, lactose, Mn^2+^, PO_4_^3−^, Zn^2+^, Na^+^, Mg^2+^, Ca^2+^, and *D*,*L*-tryptophan, which are commonly found in milk, were selected as the possible interfering substances. The influence of the above substances on the detection of hydrogen peroxide (0.2 mM) was investigated. The concentration of Fe^3+^ was 0.6 μg/mL and the concentration of the other substances was 4 mM. For real sample analysis, H_2_O_2_ in milk was detected using a standard addition method.

## 4. Conclusions

In summary, a novel ratiometric fluorescence sensing platform was constructed based on carbon dots as both luminophores and nanozymes, which realized the sensitive detection of hydrogen peroxide. CDs with peroxidase activity were prepared with a one-step hydrothermal method using *L*-histidine as an inexpensive precursor. The synthesis method was simple, green, and of a low cost. CDs had bright blue fluorescence and peroxidase-mimicking activity. The catalytic mechanism and enzymatic parameters of CD nanozymes were investigated. It was proven that CDs had good affinity for both OPD and H_2_O_2_ substrates. From the fluorescence resonance energy transfer (FRET) phenomenon between CDs and DAP, the oxidation product of OPD was confirmed. CDs catalyzed H_2_O_2_ to generate •O^2−^, which oxidized OPD to generate DAP. The FRET between CDs and DAP resulted in a decrease in the fluorescence of CDs and an increase in the fluorescence of DAP, leading to a ratiometric fluorescence analysis system. The fabricated ratiometric fluorescence system had high detection and good selectivity in the detection of H_2_O_2_. The strategy using CDs as both luminophores and nanozymes could provide new ideas for nanozyme-based sensing systems and the construction of ratiometric fluorescence sensors.

## Figures and Tables

**Table 1:** Comparison between performance of different nanozymes for H_2_O_2_ detection.

Nanozmye	Detection Method	Linear Range (μM)	LOD (μM)	Ref.
*Fe/CuSn(OH)_6_*	Colorimetric	30–1000	9.5	[51]
*Fe Pt/AuHNPs*	Colorimetric	20–700	12	[52]
*CNF/FeCDs*	Colorimetric	6–42	0.93	[53]
*Mesoporous MnCo_2_O_4_*	Colorimetric	500–120,000	200	[47]
*Ru/C_3_N_4_*	Fluorescence	0.2–1000	0.05	[49]
*N,Zn/CDs*	Fluorescence	10–70	0.008	[54]
*CDs*	Fluorescence	5–200	0.42	This work

Fe/CuSn(OH)_6_—iron-doped CuSn(OH)_6_ microspheres; Fe Pt/AuHNPs—FePt-Au ternary metallic hybrid nanoparticles; CNF/FeCDs—Fe-doped carbon dots immobilized on cellulose nanofibrils; Ru/C_3_N_4_—ruthenium ion-complexed carbon nitride; N,Zn-CDs—zinc and nitrogen co-doped multifunctional carbon dots; CDs—carbon dots.

**Table 2:** Determination of H_2_O_2_ in milk.

Sample ^a^	Spiked (μM)	Found (μM)	Recovery (%)	RSD (%)
Milk ^a^	1.0	0.98	98	2.2
	10	9.7	97	2.9
	100	102	102	1.1

^a^ Samples were diluted 10 times. The indicated concentrations were obtained after dilution.

## Data Availability

The data presented in this study are available on request from the corresponding author.
